# The mediating effect of the amygdala-frontal circuit on the association between depressive symptoms and cognitive function in Alzheimer’s disease

**DOI:** 10.1038/s41398-024-03026-3

**Published:** 2024-07-23

**Authors:** Yang Du, Shaowei Zhang, Qi Qiu, Yuan Fang, Lu Zhao, Ling Yue, Jinghua Wang, Feng Yan, Xia Li

**Affiliations:** 1 Department of Geriatric Psychiatry, Shanghai Mental Health Center, Shanghai Jiao Tong University School of Medicine, Shanghai, China; 2 Department of Psychiatry, the First Affiliated Hospital, Zhejiang University School of Medicine, Hangzhou, China

**Keywords:** Learning and memory, Diagnostic markers

## Abstract

Depressive symptoms occur commonly in Alzheimer’s disease (AD). Although abnormalities in the amygdala-frontal circuit have been linked to emotional dysregulation and cognitive impairment, the neurological basis underlying these associations in AD patients with depressive symptoms (ADD) is unclear. We aimed to investigate the relationship between the amygdala-frontal circuit and depressive symptoms and cognitive function in ADD. We recruited 60 ADD, 60 AD patients without depressive symptoms (ADND), and 60 healthy controls (HC). Functional connectivity (FC) maps of the bilateral amygdala were compared. Fractional anisotropy (FA) of the amygdala-frontal circuit connected by the uncinate fasciculus (UF) was calculated using automated fiber quantification (AFQ). In addition, mediation analysis was performed to explore the effects of the amygdala-frontal circuit on the relationship between depressive symptoms and cognitive function. We found decreased bilateral amygdala FC with the inferior frontal gyrus (IFG) in the ADD group compared to the ADND and HC groups. Moreover, FA in the left frontal UF (nodes 64–97) was significantly lower in the ADD group than ADND group. Notably, amygdala-based FC with IFG and the left frontal UF FA mediated the relationship between depressive symptoms and cognitive function in ADD, with mediating effects ranging between 15 and 18%. Our study is the first to demonstrate the mediating effect of functional and microstructural abnormalities in the amygdala-frontal circuit in ADD. The findings suggest that the amygdala-frontal circuit may underlie emotional dysregulation in ADD, providing potential targets for treatment strategies.

## Introduction

Alzheimer’s disease (AD) is a neurodegenerative disorder with the primary clinical manifestation of cognitive impairment [1]. Moreover, up to 80% of patients with AD also exhibit behavioral and psychological symptoms of dementia (BPSD) [2]. Depressive symptoms are among the most common BPSD in AD patients, with a prevalence ranging from 37 to 46% [3]. Evidence that depressive symptoms are associated with more impaired cognitive function and faster subsequent cognitive decline [4]. Considering the high prevalence and poor prognosis of depressive symptoms in AD, the pathophysiological mechanisms of depressive symptoms and cognitive function need to be explored. The amygdala-frontal circuit involves emotional dysregulation and cognitive integration, the microstructural and functional abnormalities of this circuit are strongly associated with both depression and AD [5]. However, it is not clear how functional and microstructural alterations in the amygdala-frontal circuit contribute to depressive symptoms in AD.

Resting-state functional magnetic resonance imaging (rs-fMRI) is a non-invasive technique to examine the neural connectivity of the human brain [6]. Seed-based functional connectivity (FC) could reflect stimulus-independent temporal correlations of neuronal activity patterns between the seed with other spatially segregated brain areas [7]. One previous fMRI study found that AD patients with depressive symptoms (ADD) showed decreased amygdala-based FC with medial prefrontal cortex and inferior frontal gyrus (IFG) compared to AD patients without depressive symptoms (ADND) [8], which may reflect impaired amygdala-frontal circuit leading to abnormal emotional regulation in ADD. Current neuroimaging studies have found that abnormal amygdala FC with brain regions in ADD patients is mostly concentrated on the frontal lobe, suggesting the potential effect of the amygdala-frontal circuit in ADD [9]. Moreover, one previous study showed that amygdala FC with the middle frontal gyrus in ADD patients mediated the relationship between depressive symptoms and cognitive function in AD, suggesting that the amygdala-frontal circuit may be a mediator between depression and cognition [10]. However, the above study did not compare amygdala-based FC between ADD and ADND patients, and therefore amygdala-based FC for mediation analyses was not an ADD-specific alteration. Therefore, amygdala-specific FC alterations in ADD and their potential effect on the relationship between depressive symptoms and cognitive function in ADD have not been investigated.

Diffusion tensor imaging (DTI) is a well-established neuroimaging technique intended to infer the properties of structural brain connectivity in vivo [11]. The uncinate fasciculus (UF) is the central white matter tract connecting the frontal cortex to the amygdala involved in emotion regulation and episodic memory [12]. A previous DTI study demonstrated greater white matter microstructural abnormalities of the UF in ADD patients compared to ADND patients, suggesting that dysfunction of the amygdala-frontal circuit might contribute to the pathophysiology of depressive symptoms in AD [13]. Moreover, several DTI studies found that fractional anisotropy (FA) in the UF was significantly lower in AD patients and associated with cognitive function [14, 15]. One DTI study showed that the integrity of the left UF correlated with the severity of depressive symptoms and cognitive function in patients with depression [16]. However, the relationship between the white matter microstructure of the UF and depressive symptoms and cognitive function is not clear in ADD.

Notably, compared to averaging diffusion metrics for the entire UF, automatic fiber quantification (AFQ) can automatically quantify diffusion metrics at equivalent locations along the trajectory to identify local variations [17]. To date, no study has provided a detailed description of the abnormal trajectory of the UF in patients with ADD. Understanding the complete profile of the trajectory of the UF could provide specific biomarkers for ADD and lay the basis for novel therapeutic interventions. Moreover, neuroimaging studies have found decreased functional and structural connectivity of the amygdala-frontal circuit in patients with depression, suggesting the essential effect of the amygdala-frontal circuit in the neuropathophysiology of depression [18]. However, there are no multimodal studies combining fMRI with DTI methods to explore structural and functional abnormalities of the amygdala-frontal circuit in patients with ADD.

In this study, first, we compared amygdala-based FC among ADD, ADND, and HC groups. Second, we evaluated bilateral UF by quantifying diffusion properties at multiple nodes using the AFQ method among the three groups. Finally, we conducted correlation and mediation analyses to explore whether functional and microstructural abnormalities might mediate the correlation between depressive symptoms and cognitive function. We hypothesized that there were microstructural and functional abnormalities in the amygdala-frontal circuit and mediated the relationship between depressive symptoms and cognition in ADD.

## Methods

### Study participants

Based on previous studies, a total sample size of 180 had a relatively good positive prediction rate, sensitivity, and reproducibility in fMRI studies [19]. Therefore, we recruited 60 patients with ADD and 60 patients with ADND from the Shanghai Action of Dementia Prevention for the Elderly (SHAPE) cohort, and 60 HCs were recruited from the community matched for age, sex, and years of education. AD patients were diagnosed by two experienced geriatric psychiatrists from the Shanghai Mental Health Centre according to the National Institute on Aging-Alzheimer’s Association guidelines (NIA-AA) [20]. We used the Mini-mental State Examination (MMSE) scale to assess the severity of cognitive impairment in AD, and the score on the MMSE scale of no more than 24 [21]. We used the provisional diagnostic criteria for depression in AD to diagnose depression [22]. To eliminate the effect of previous episodes of depression in youth on brain structure and function, we excluded AD patients with a previous diagnosis of depression in their youth.

The 30-item Geriatric Depression Scale (GDS-30) was used to evaluate the severity of depressive symptoms in AD patients [23]. Moreover, higher scores of the GDS indicate greater severity of depressive symptoms, and a cutoff score of 10 has acceptable discriminant validity for clinical depression [24]. Therefore, the inclusion criteria for ADD patients included meeting a GDS-30 score of no less than 10, whereas ADND patients required a GDS-30 score of less than 10. Notably, the assessment of depressive symptoms is more significant in mild AD [25], therefore our study only included mild AD patients with the Clinical Dementia Rating (CDR) score = 0.5 or 1.

The inclusion criteria for HC subjects were as follows: (1) no neurological disorders; (2) no psychiatric disorders; (3) MMSE score ≥27; and (4) a GDS score lower than 10. The exclusion criteria for subjects were as follows: (1) other types of dementia; (2) history of depression, schizophrenia, bipolar disorder, and other severe mental illnesses; (3) comorbid with systemic diseases that may cause cognitive impairment; (4) people with contraindications to MRI scanning; and (5) unable to complete the neuropsychological assessments. This retrospective study was approved by the Ethics Committee of Shanghai Mental Health Centre, School of Medicine, Shanghai Jiao Tong University, and written informed consent was obtained from all participants. The study involving human subjects, human material, or human data is in accordance with the Declaration of Helsinki.

### Image data acquisition

All imaging data were obtained on a 3-Tesla MRI scanner (Siemens, Munich, Germany) with an 8-channel phased array head coil. Each subject underwent consecutive structural MRI, fMRI, and DTI scans on the same MRI scanner with all identical scanning parameters. T1-weighted images were acquired using a magnetization-prepared rapid gradient echo imaging (MPRAGE) sequence with the following parameters: repetition time (TR)/echo time (TE) = 2530 ms/3.5 ms, flip angle = 9°, matrix size = 256 × 256, voxel size = 1 × 1 × 1 mm^3^, slice thickness = 1.2 mm. The fMRI data were obtained with the gradient echo-planar imaging sequence with the following parameters: TR/TE = 2000 ms/30 ms, flip angle =90°, matrix size = 74 × 74, voxel size = 3 × 3 × 3 mm^3^, slice thickness = 3 mm. The entire fMRI scan lasted 8 min and generated 200 volumes. The DTI MRI data was collected using a single-shot spin-echo echo-planar imaging sequence with 64 noncollinear directions (b = 1000 s/mm^2^) and a reference image without a diffusion weighting gradient (b = 0). The parameters of DTI data acquisition were as follows: TR/TE = 5620 ms/106 ms; matrix size = 126 × 126; voxel size = 2 × 2 × 2 mm^3^, slice thickness = 2 mm, 74 slices. Axial sections were performed parallel to the anterior–posterior commissural line to cover the whole brain. The total DTI scan lasted 6 min.

### Image data preprocessing

The rs-fMRI data preprocessing was performed with the Data Processing Assistant for Resting-State fMRI software (DPARSF, http://www.restfmri.net) to perform the image preprocessing. We discarded the first ten time points and conducted slice timing correction. We used the Friston 24-parameter model to minimize the effects of head motion. All subjects were under the threshold of spatial movement in any direction <2 mm or 2°, and the mean framewise displacement was <0.5 mm. Next, the images were normalized to the standard Montreal Neurological Institute (MNI) space using the DARTEL tool, and each voxel was spatially resampled to 3 × 3 × 3 mm^3^. The processed images were smoothed with a full width at half maximum (FWHM) of 6 mm. Moreover, removing linear trends and temporal band-pass filtering (0.01–0.08 Hz) was conducted. Finally, nuisance covariates (head motion parameters, cerebrospinal fluid, and white matter) were regressed to reduce the effects of non-neuronal BOLD fluctuations.

DTI data were preprocessed using the toolbox of FMRIB Software Library (http://www.fmrib.ox.ac.uk/fsl). The preprocessing steps were as follows. For each DTI data, all diffusion-weighted images were affinely co-registered to the b0 image to correct for eddy current-induced distortion and subtle head motion. The transformation matrix was extracted for each volume to adjust the direction of the diffusion gradient. A brain mask was created by removing extracerebral tissue from the b0 image using the BET command with a fractional intensity threshold of 0.2. The FMRIB Diffusion Toolbox (FDT) was used to fit a tensor model to the image data for each voxel, and FA, mean diffusivity (MD), radial diffusivity (RD), and axial diffusivity (AxD) metrics were calculated.

### Seed-based functional connectivity analysis

The Resting-State fMRI Data Analysis Toolkit (REST) was used to perform seed-based rs-FC analyses [26]. The bilateral amygdala was created using the SPM Anatomy Toolbox [27]. First, we extracted the time series averaged across all voxels within each seed. Then, we conducted Pearson’s correlation coefficients between the time series of each seed and other voxels across the whole brain. The voxel-wise Pearson’s correlation coefficients were z-scored using Fisher’s Z transform for further statistical analyses.

### Automatic fiber quantification analysis

The AFQ toolkit package (https://github.com/yeatmanlab/AFQ) was used to identify the trajectory of bilateral UF, which implemented quantifying diffusion measures along each fiber tract. Firstly, whole-brain deterministic fiber tracking was performed. Tracking terminated when the FA value was lower than 0.2 or the minimum angle between the last path segment and the next step was greater than 30°. We performed fiber tract segmentation using the waypoint ROI-based procedure [28]. Fiber refinement was based on the fiber tract probability maps [29], and fiber cleaning was according to the outlier elimination algorithm [30]. After identifying the bilateral UF tracts, each fiber tract was sampled along 100 isometric nodes, and diffusion measurement values were extracted along the UF.

### Statistical and mediation analysis

We tested for normality and homogeneity of variance for all data. We compared the demographic and clinical characteristics of subjects from different groups. Age and education level of the ADD, ADND, and HC groups were compared using a one-way analysis of variance (ANOVA), and sex distribution was compared using a chi-square test. Moreover, differences in clinical data, including MMSE, CDR, and GDS scores, were assessed using ANOVA, and post-hoc tests were performed on any two groups. Controlling for age, gender, and education, we performed partial correlation analyses between depressive symptoms measured by the GDS-30 score and cognitive function measured by the MMSE score in patients with ADD.

ANOVA design model was used to compare significant differences in the FC maps among the ADD, ADND, and HC groups, with age, sex, and education as covariates. A non-parametric approach using permutation tests tends to be more applicable for controlling the false positive rate [31]. Therefore, voxel-wise comparisons were performed using a non-parametric permutation test with 5000 permutations, and multiple comparisons were corrected using the threshold-free cluster enhancement (TFCE) method and thresholded at *P* < 0.05. Brain regions with significant differences among groups were extracted as a mask, and post-hoc two-sample *t*-tests were performed within the mask to examine differences between each group pair. Mean z-score FC values were extracted from brain regions with significant differences between the ADD and ADND groups. In addition, we performed partial correlations using age, gender, and education as covariates to explore the relationships between amygdala-based FC and clinical profiles, including MMSE and GDS scores.

Between-group differences in diffusion measurement values, including FA, MD, AxD, and RD at uniform intervals of 1–100 nodes along the UF in the ADD, ADND, and HC groups, were assessed using ANOVA, followed by post-hoc comparisons using the Turkey test. Given the potential correlation between neighboring segments of the UF, the Bonferroni correction is excessively strict and unfit for our analysis. Therefore, we chose the false discovery rate (FDR) correction to the total number of statistical tests (100 nodes × 2 tracts) [32], and the significance level was set at *P* < 0.05 (two-tailed). Moreover, we used partial correlation analyses to explore the relationship between significantly abnormal diffusion values of the UF and cognitive function and depressive symptoms in ADD, controlling for age, gender, and education.

We conducted mediation analyses using SPSS PROCESS (Hayes, 2013) to examine whether amygdala-based FC and diffusion values of the UF mediated the between depressive symptoms and cognitive function in patients with ADD, using age, gender, and education as covariates. A bootstrap analysis was conducted on 10,000 samples, calculating 95% CIs and testing for the significance of mediation and moderation effects, with 95% CIs that did not include zero being considered significant (Hayes, 2018).

## Results

### Demographic and clinical characteristics

The demographic and clinical characteristics of the ADD, ADND, and HC groups are shown in Table 1. A total of 180 individuals, including 60 ADD patients, 60 ADND patients, and 60 HC completed this study. There were no significant differences in age (*P* = 0.82), sex (*P* = 0.75), and education (*P* = 0.87) among the three groups (Table 1). ADD and ADND groups showed significantly higher MMSE and CDR scores than the HC group (*P* < 0.001), and there was no significant difference in MMSE and CDR scores between ADD and ADND groups. The ADD group showed significantly higher GDS scores than the ADND and HC groups (*P* < 0.001).

**Table 1:** Demographic data and clinical measures among three groups.

Groups	ADD	ADND	HC	*F*-value	*p*-value
Subjects	60	60	60	-	-
Age	71.18 ± 7.75	71.93 ± 6.84	71.72 ± 4.20	0.21	0.82
Sex (F/M)	40/20	36/24	38/22	5.73	0.75
Education	12.23 ± 3.12	12.02 ± 2.63	12.30 ± 2.94	0.16	0.87
MMSE	18.58 ± 4.06	18.60 ± 3.71	28.30 ± 1.22	149.10	<0.001^b,c^
GDS-30	13.15 ± 3.37	1.98 ± 1.64	1.60 ± 1.39	445.19	<0.001^a,c^
CDR	0.68 ± 0.24	0.69 ± 0.25	0	197.96	<0.001^b,c^

*ADD* Alzheimer’s disease patients with depressive symptoms, *ADND* Alzheimer’s disease patients without depressive symptoms, *HC* healthy controls, *MMSE* Mini-Mental State Examination, *GDS-30* the Geriatric Depression Screening Scale with 30 items, *CDR* Clinical Dementia Rating Scale.

a–c: post-hoc analysis following one-way analysis of variance (a: ADD vs. ADND, b: ADND vs. HC, and c: ADD vs. HC).

### Functional connectivity results

**Fig. 1:**
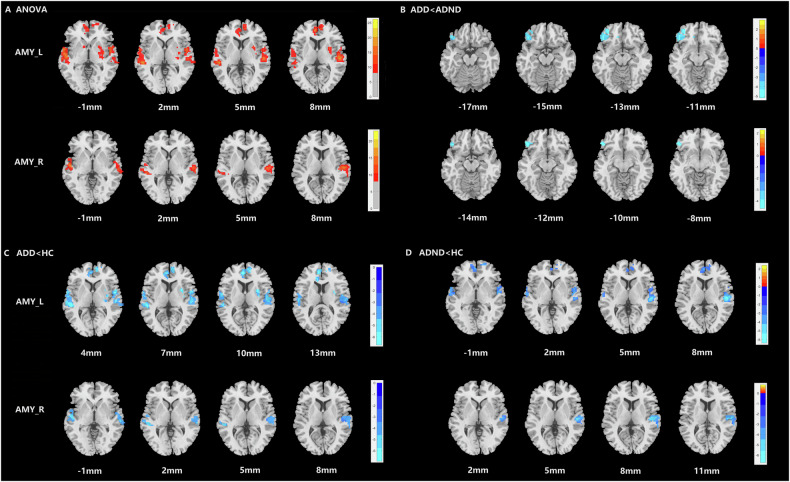
Group comparison of FC between the left and right amygdala and whole brain in ADD and ADND and HC groups. **A** ANOVA, **B** ADD<ADND, **C** ADD<HC, **D** ADND<HC. The color bar represents the range of F or T values. ADD AD patients with depressive symptoms, ADND AD patients without depressive symptoms, HC healthy controls, AMY_L the left amygdala, AMY_R the right amygdala.

The ANOVA showed significant differences between the bilateral amygdala and bilateral median cingulate and paracingulate gyrus (MCC), bilateral superior temporal gyrus (STG), right supplementary motor area (SMA), right parahippocampal gyrus (PHG), and left IFG among the ADD, ADND, and HC groups (Fig. 1A; Table 2). Compared with ADND patients, ADD patients demonstrated decreased bilateral amygdala FC with left IFG (Fig. 1B; Table 2). Regarding the left amygdala, patients with ADD exhibited decreased left amygdala FC with bilateral MCC, bilateral STG, right SMA, right PHG, and left IFG compared with HCs. Regarding the right amygdala, decreased FC existed between the right amygdala and bilateral MCC, bilateral STG, bilateral PHG, and left IFG in ADD patients compared with HCs (Fig. 1C; Table 2). In ADND patients, left amygdala FC with bilateral MCC and bilateral STG were decreased, and right amygdala FC with bilateral MCC and right STG, and bilateral PHG were also decreased compared with HCs (Fig. 1D; Table 2).

**Table 2:** Brain regions with significant differences in amygdala-based FC among ADD, ADND, and HC groups.

Seed region	Area	L/R	Voxels	MNI X Y Z			F/T-value
				X	Y	Z	
Left amygdala							
ANOVA	MCC	L/R	1553	−12	−24	33	26.20
	STG	L	329	−51	−30	3	19.71
		R	292	54	−18	9	18.27
	IFG	L	178	−36	27	−21	14.87
	SMA	R	239	6	−9	63	14.06
	PHG	R	207	30	−21	−18	13.13
ADD < ADND	IFG	L	125	−48	27	−9	−4.58
ADD < HC	MCC	L/R	1525	9	−18	33	−6.51
	SMA	R	225	6	−9	63	−6.35
	STG	L	326	−51	−30	3	−6.71
		R	291	51	3	0	−5.94
	PHG	R	201	30	−21	−18	−5.80
	IFG	L	150	−36	27	−21	−6.46
ADND < HC	MCC	L/R	932	−12	24	33	−6.39
	STG	R	192	54	−21	9	−5.70
		L	52	−60	−9	3	−3.98
Right amygdala							
ANOVA	MCC	L/R	587	−9	−36	24	23.01
	IFG	L	129	−30	21	−21	20.15
	PHG	R	139	18	−12	−15	17.23
		L	194	−27	−9	−30	16.68
	STG	L	136	−54	−9	−3	16.64
		R	205	54	−18	9	16.09
ADD < ADND	IFG	L	42	−51	33	−9	−4.67
ADD < HC	MCC	L/R	584	12	−15	33	−5.99
	STG	L	135	−54	−9	−3	−5.92
		R	200	63	−21	0	−4.73
	PHG	L	194	−27	−12	−27	−6.17
		R	137	36	−18	−24	−5.65
	IFG	L	125	−30	21	−24	−6.94
ADND < HC	MCC	L/R	586	−9	−36	24	−6.72
	STG	R	159	54	−18	9	−5.57
	PHG	L	53	−33	−18	−33	−4.91
		R	66	36	−18	−36	−4.63

*ADD* AD patients with depressive symptoms, *ADND* AD patients without depressive symptoms, *HC* healthy control, *IFG* inferior frontal gyrus, *MCC* median cingulate and paracingulate gyrus, *STG* superior temporal gyrus, *SMA* supplementary motor area, *PHG* parahippocampal gyrus.

### Automatic fiber quantification results

**Fig. 2:**
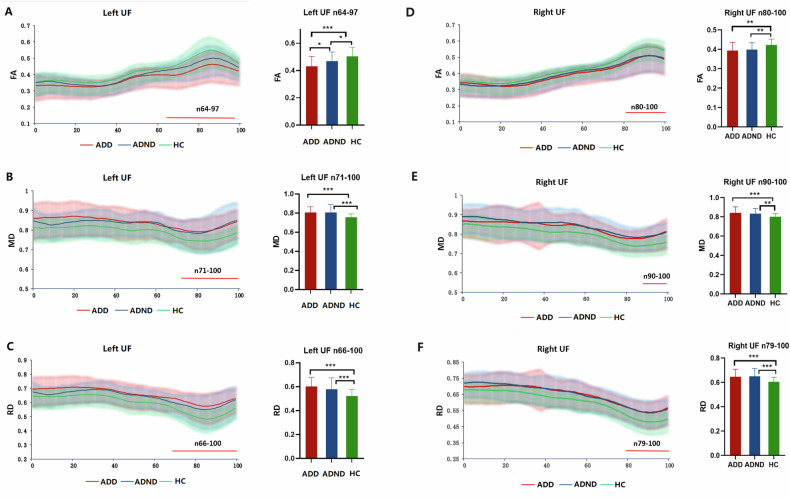
The plots of FA, MD, and RD profiles of significant alterations in UF among ADD, ADND, and HC groups (FDR correction, *p* < 0.05, green for HC, blue for ADND, and red for ADD). **A** FA of left UF, **B** MD of left UF, **C** RD of left UF, **D** FA of right UF, **E** MD of right UF, **F** RD of right UF. The solid line represents the mean and the shaded area represents the standard deviation. The red bars under the profile indicate the regions of significant difference among the three groups. The histograms show statistically significant differences in FA, MD, and RD of UF nodes among the ADD, ADND, and HC groups. FA fractional anisotropy, RD radial diffusivity, MD mean diffusivity, UF uncinate fasciculus.

Significant differences in FA in the left frontal UF in nodes 64–97 were found among the three groups (*P*_FDR-corrected_ < 0.001). Post-hoc analyses showed that FA in the left frontal UF (nodes 64–97) in the ADD group was significantly lower compared with the ADND (*P*_FDR-corrected_ = 0.019) and HC groups (*P*_FDR-corrected_ < 0.001), and FA in the left frontal UF (nodes 64–97) in the ADND group was significantly lower compared with the HC group (*P*_FDR-corrected_ = 0.020) (Fig. 2A). In addition, there was a significant difference in MD in left frontal UF in nodes 71–100 among the three groups (*P*_FDR-corrected_ < 0.001). Post-hoc analyses showed that patients with ADD and ADND (*P*_FDR-corrected_ < 0.001) showed significantly lower FA in the left frontal UF in nodes 71–100 compared with the HC group (Fig. 2B). However, there was no significant difference in MD of left UF in ADD patients compared to ADND patients. Moreover, significant differences among the three groups were found in the RD of the left frontal UF in nodes 66–100 (*P*_FDR-corrected_ < 0.001). The ADD and ADND patients showed significantly lower FA in the left frontal UF (nodes 66–100) compared to the HC group (*P*_FDR-corrected_ < 0.001) (Fig. 2C). Post-hoc analysis revealed no significant difference in the MD of left UF in ADD patients compared with ADND patients. Furthermore, there was no significant difference between the three groups at any of the nodes in AxD in the left UF.

For the right frontal UF, the difference of FA in nodes 80–100 among the three groups was significant (*P*_FDR-corrected_ = 0.003). Post-hoc analyses showed significantly lower FA in the right frontal UF (nodes 80–100) in the ADD group (*P*_FDR-corrected_ = 0.002) and ADND group (*P*_FDR-corrected_ = 0.005) compared to the HC group (Fig. 2D). In addition, a significant difference in MD in right frontal UF in nodes 90–100 was observed among the three groups (*P*_FDR-corrected_ = 0.002). Post-hoc analyses showed that ADD patients (*P*_FDR-corrected_ < 0.001) and ADND patients (*P*_FDR-corrected_ = 0.001) revealed significantly lower FA in the right frontal UF in nodes 90–100 compared with the HC group (Fig. 2E). Furthermore, there was a significant difference in the RD of the right frontal UF in nodes 79–100 among the three groups (*P*_FDR-corrected_ < 0.001). The ADD and ADND patients (*P*_FDR-corrected_ < 0.001) showed significantly lower FA in the right frontal UF (nodes 79–100) compared to the HC group (Fig. 2F). However, there was no significant difference in MD and RD of right UF in ADD patients compared to ADND patients. Moreover, no significant difference was observed between the three groups at any of the nodes in AxD in the right UF.

### Correlation and mediation analysis results

**Fig. 3:**
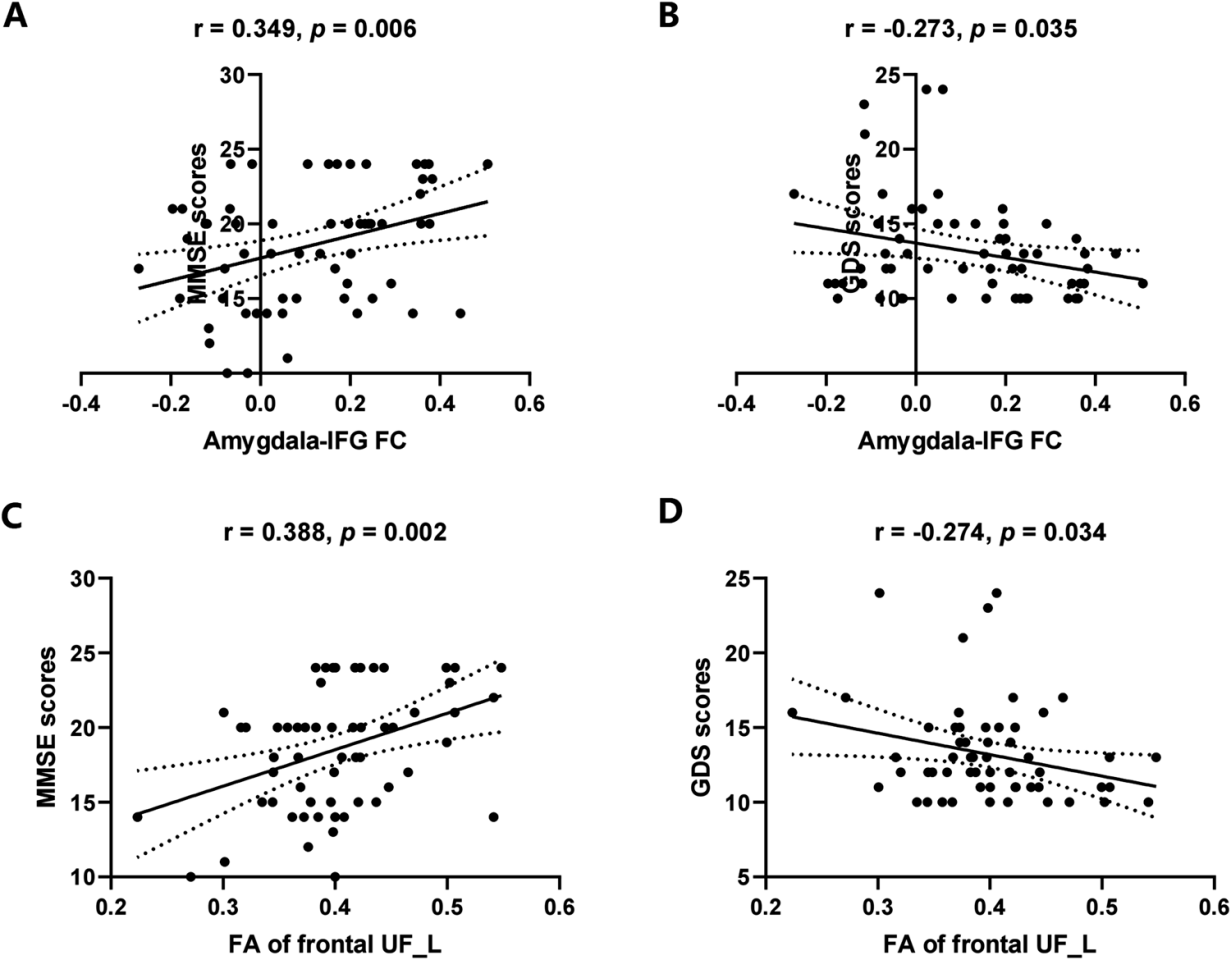
The relationship between amygdala-based structural and functional alterations and depressive symptoms and cognitive function in ADD. **A** Amygdala-IFG FC associated with MMSE scores, **B** amygdala-IFG FC associated with GDS scores, **C** FA of frontal UF_L associated with MMSE scores, **D** FA of frontal UF_L associated with GDS scores. IFG inferior frontal gyrus, FC functional connectivity, UF uncinate fasciculus, FA fractional anisotropy, GDS Geriatric Depression Scale, MMSE Mini-Mental State Examination.

We found that depressive symptoms were negatively correlated with cognitive function in ADD patients (*r* = −0.445, *P* < 0.001). In addition, FC between the left amygdala and left IFG in ADD patients was positively correlated with MMSE scores (*r* = 0.349, *P* = 0.006) and negatively correlated with GDS scores (*r* = −0.273, *P* = 0.035) (Fig. 3A, B). The FA in nodes 64–97 of the left frontal UF showed positively correlated with MMSE scores (*r* = 0.388, *P* = 0.002) and negatively correlated with GDS scores (*r* = −0.274, *P* = 0.034) (Fig. 3C, D). There was no significant correlation between other abnormal metrics in the bilateral UF nodes and MMSE or GDS scores.

**Fig. 4:**
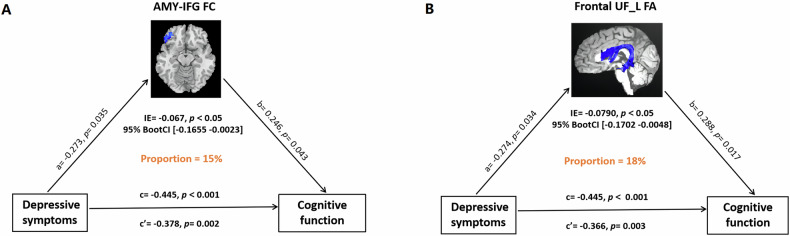
The mediation effect of amygdala-based structural and functional alterations on the influences of depression and cognition in ADD. **A** Amygdala-IFG FC mediated the relationship between depression and cognition, **B** frontal UF_L FA mediated the relationship between depression and cognition. IFG inferior frontal gyrus, FC functional connectivity, FA fractional anisotropy.

We found that FC between the left amygdala and left IFG partially mediated the relationship between depressive symptoms measured by GDS scores and cognitive function measured by MMSE scores (indirect effect = −0.0670, bootstrapped 95% CI = [−0.1655, −0.0023], *P* < 0.05) with the approximate proportion of mediation of 15% (Fig. 4A) in ADD. Moreover, our findings showed that FA in nodes 64–97 of the left UF partially mediated the relationship between depressive symptoms measured and cognitive function (indirect effect = −0.0790, bootstrapped 95% CI = [−0.1702, −0.0048], *P* < 0.05) with the approximate proportion of mediation of 18% (Fig. 4B) in ADD.

## Discussion

This is the first study to explore abnormalities in the structure-function of the amygdala-frontal circuit in ADD patients. We found that ADD patients showed decreased amygdala-based FC with left IFG and lower FA in left frontal UF compared to ADND patients and mediated the relationship between depressive symptoms and cognitive function in ADD. The mediating effects suggest that depression may contribute to cognitive impairment through functional and microstructural alternations in the amygdala-frontal circuit. The findings of the amygdala-frontal circuit provide novel insights into the neural mechanisms of ADD, indicating that the amygdala-frontal circuit might be an intervention target for the clinical treatment of ADD patients.

We found that ADD patients demonstrated decreased FC between the bilateral amygdala and left IFG compared with ADND patients. The IFG is a critical brain region involved in the process of higher-order functional neural networks of emotion recognition and cognitive modulation [33]. Previous studies have found decreased regional alterations in IFG activity in ADD patients [34]. Several studies showed that ADD patients have lower hypoperfusion of the amygdala and IFG compared to ADND patients [35], suggesting the amygdala-frontal circuit contributes to the pathophysiology of ADD. Moreover, one fMRI study has shown decreased FC between the amygdala and the IFG in ADD patients compared with ADND patients, which is consistent with our findings [8]. The decreased amygdala-IFG FC might reflect a failure of engaging high-order cortical to regulate the emotion-related limbic area of the amygdala, leading to emotional dysregulation. Our findings may provide the basis for understanding the abnormal FC patterns of the amygdala-frontal circuit underlying the pathophysiology of ADD.

We found that the amygdala FC with the IFG partially mediated the association between depression symptoms and cognitive function in AD. Several fMRI studies have found the FC between the amygdala and IFG correlates with episodic memory performance [36, 37]. One study found that depressive symptoms indirectly affect cognitive function through episodic memory [38]. Therefore, amygdala-IFG FC may influence episodic memory processing and thus mediate the process of depressive symptoms affecting cognitive dysfunction in ADD. Moreover, a recent study found that repetitive transcranial magnetic stimulation (rTMS) with the IFG significantly improved cognitive function in AD patients [39], providing a prospective target for cognitive improvement. Our findings on the mediating effect of amygdala-IFG FC provide new insights into the neural substrate links between depressive symptoms and cognitive function in AD pathology and may serve as a potential target for intervention treatments.

In this study, ADD patients showed lower FA in the left frontal UF than ADND patients. The UF is the major structural pathway connecting the amygdala with the frontal regions, which is involved in emotion regulation and cognitive processing [40]. Our finding of lower FA in the frontal UF suggests disrupted structural integrity between the frontal regions and amygdala, which might lead to emotion dysregulation in AD. There is evidence that the disconnection of white matter fiber may provide a structural basis for abnormal functional connectivity of its connecting brain regions [41], explaining the consistency of our findings of structural and functional disturbances in the amygdala-frontal circuit of ADD patients. Previous DTI studies have reported that lower FA in the left UF were found in AD and depression patients [42, 43]. However, previous studies focused on abnormalities in the entire UF, which averaged over the entire tract and may mask potentially significant local variations. Our study is the first to use the AFQ method to explore the differences in the focal abnormality of the UF in ADD and ADND patients. We indicated that decreased FA was concentrated in the frontal part of the left UF in ADD patients, suggesting that it may be a characteristic neuroimaging biomarker of ADD.

We demonstrated that the left frontal UF FA mediated the relationship between depressive symptoms and cognitive function in ADD. Several DTI studies showed that FA of the left UF was lower in AD patients and correlated with cognitive performance [44, 45], suggesting that disrupted microstructure of the left UF is a promising biomarker for monitoring disease progression. Moreover, one DTI study showed a positive trend between verbal fluency scores and FA values of left UF in AD patients [46]. The existing evidence has shown that lower FA of the UF is associated with higher amyloid burden in preclinical AD [47], indicating microstructural changes in the UF are accompanied by pathological accumulation in AD. Moreover, one previous study has demonstrated that amyloid pathology might partially mediate the influences of depressive symptoms on cognitive impairments [48]. Therefore, the microstructure of the UF may interact with amyloid pathology, thus mediating the relationship between depressive symptoms and cognitive function in ADD. The mediating effect of the left frontal UF FA provides a better understanding of the relationship between depressive symptoms and cognitive impairments through microstructural alterations, suggesting that the left UF may be a valuable target for intervention therapy.

In our study, left amygdala FC with the left IFG and the left frontal UF FA mediated the relationship between depressive symptoms and cognitive function in ADD. Interestingly, significant results were clustered in the left amygdala-frontal circuit. Neuroimaging studies have revealed lateralized effects of amygdala function during emotional processing [49]. Different from the automatized role of the right amygdala during emotional processes, the left amygdala is involved in continuous and voluntary emotional appraisal processes [50]. Therefore, we speculated that the left amygdala FC pattern may consistently influence mood and cognition in ADD patients, thus acting as a mediator of the relationship. A previous study showed that the WM microstructure of the left UF was associated with socio-emotional function [51]. Moreover, neuroimaging evidence has demonstrated the presence of a relatively left-sided dominant abnormality in the UF, which is critical for emotional processing and cognitive, sensory, and motor function [52]. In our study, an AFQ analysis of bilateral UF was performed, and FA was significantly lower in ADD patients compared to ADND patients in the left frontal UF only, which also supports the functional lateralization of the left UF.

There are some limitations in this study. First, our study was a cross-sectional design, and depressive symptoms and cognitive function in AD patients tend to change over time. The lack of dynamic assessment of disease symptoms makes it difficult to provide a causal relationship between depressive symptoms, cognitive function, and the amygdala-frontal circuit. Longitudinal studies are necessary to be designed to explore the underlying mechanisms. Second, the amygdala is a heterogeneous structure containing structurally and functionally discrete subregions, which may have distinct cytoarchitecture and functional connectivity patterns [53]. In future studies, clarifying the alterations of amygdala subregional functional networks may facilitate understanding the neurobiological mechanisms of ADD.

Our study is the first to combine structural and functional neuroimaging in ADD patients, demonstrating functional-structural impairment of the amygdala-frontal circuit. Notably, we found the mediating effect of the amygdala-frontal function and microstructure on the relationship between depressive symptoms and cognitive function in ADD. These findings provide the basis for the complex and interacting relationships between the amygdala-frontal circuit and emotion and cognition, indicating that the amygdala-frontal circuit may be a potential target for therapeutic intervention.

## Data Availability

The data that support the findings of this study are available from the corresponding author upon reasonable request.
